# An Assessment of the Efficacy and Safety of CROSS Technique with 100% TCA in the Management of Ice Pick Acne Scars

**DOI:** 10.4103/0974-2077.69020

**Published:** 2010

**Authors:** Deepali Bhardwaj, Niti Khunger

**Affiliations:** *Department of Dermatology and STD, VM Medical College and Safdarjang Hospital, New Delhi – 110 088, India*

**Keywords:** Chemical reconstruction of skin scars, ice pick acne scars, trichloroacetic acid 100%

## Abstract

**Background:**

Chemical reconstruction of skin scars (CROSS) is a technique using high concentrations of trichloroacetic acid (TCA) focally on atrophic acne scars to induce inflammation followed by collagenisation. This can lead to reduction in the appearance of scars and cosmetic improvement.

**Aims:**

The aim of this pilot study is to investigate the safety of the CROSS technique, using 100% TCA, for atrophic ice pick acne scars.

**Settings and Design:**

Open prospective study.

**Material and Methods:**

Twelve patients with predominant atrophic ice pick post acne scars were treated with the CROSS technique, using 100% TCA, applied with a wooden toothpick, at two weekly intervals for four sittings. Efficacy was assessed on the basis of the physician’s clinical assessment, photographic evaluation at each sitting and patient’s feedback after the fourth treatment, and at the three-month and six-month follow-up period, after the last treatment.

**Results:**

More than 70% improvement was seen in eight out of ten patients evaluated and good results (50 – 70% improvement) were observed in the remaining two patients. No significant side effects were noted. Transient hypopigmentation and hyperpigmentation was observed in one patient each. Physician’s findings were in conformity with the patient’s assessment. Three months after the last treatment, one patient noted a decrease in improvement with no further improvement even at the six-month follow-up period.

**Conclusion:**

The CROSS technique with 100% TCA is a safe, efficacious, cost-effective and minimally invasive technique for the management of ice pick acne scars that are otherwise generally difficult to treat. In few patients the improvement may not be sustained, probably due to inadequate or delayed collagenisation.

## INTRODUCTION

Acne vulgaris is a common disorder in the adolescent age group and unfortunately, post acne facial scarring is common. Scarring in acne as proposed by Jacob *et al*,[1] is of three types: ice pick, rolling and boxcar types, and is considered to be related both to the severity of the acne lesions and also delay in the treatment. Ice pick scars are narrow, less than 2 mm wide, punctiform deep scars, with the opening generally wider than the deeper infundibulum, forming a ‘v’ shape. Rolling scars are depressed, distensible scars, with gentle sloping edges, whereas, boxcar scars are shallow or deep, punched out ‘u’ shaped scars that may be round, polygonal or linear. As acne scars are polymorphic and different type of scars can occur in the same patient, the treatment has to be designed according to the type of scars. There is no single effective technique for the various types of scars and multiple techniques have to be used. There are multiple treatment options for atrophic acne scars. Subcision, dermabrasion, nonablative lasers and laser resurfacing are useful for shallow atrophic scars, but inadequate for deeper scars such as ice pick scars that can extend deep into the dermis and subcutaneous tissue. Punch excision techniques or punch grafting are preferred methods for deep scars, which may then be combined with resurfacing techniques. In addition, laser resurfacing or dermabrasion techniques are associated with considerable morbidity and invariably with prolonged downtime.[2] Hence the search for a treatment that is effective and safe.

Trichloroacetic acid (TCA) is an established peeling agent, which is mostly used for superficial as well as medium depth peel.[3,4] The peel depth varies according to its concentration^3^; higher concentrations can reach deeper depths and modify the deeper scars. Being a self-neutralizing agent it does not get absorbed in the circulation, hence high concentrations can be safely used. In order to maximise the effects and overcome complications such as scarring and pigmentary changes that can occur with deep chemical peels, focal application of TCA, restricted to the atrophic scars has been conducted. The technique called CROSS was first described by Lee *et al*.[5] In this technique a high concentration of TCA is applied focally, by pressing a sharpened wooden applicator. hard on the entire depressed area of the ice pick scar. A frosted appearance on each scar is produced. Healing is more rapid and associated with a lower complication rate as the adjacent, normal tissue and adnexal structures are spared.

The aim of this pilot study was to assess the safety and efficacy of this new technique with the maximum effective concentration of TCA (100%) in the management of ice pick acne scars.

## MATERIALS AND METHODS

**Figure 1:**
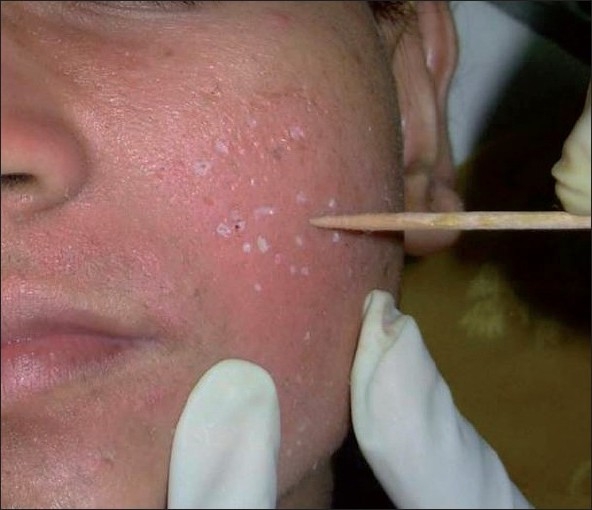
Application of TCA 100% with a wooden toothpick, keeping the skin stretched till frosting occurs

Twelve patients, ten females and two males, age ranging from 14 to 42 years, with predominantly ice pick acne scarring were included. Patients with active inflammatory lesions, keloidal tendency or infections such as herpes labialis and those on systemic isotretinoin were excluded from the study. An informed explanatory consent was taken from all patients before initiation of the therapy. They were subjected to a common protocol of management. Patients were initially primed for two weeks with tretinoin 0.025% cream at night and a sunscreen containing avobenzone, octinoxate and 4% hydroquinone in morning before starting the 100% TCA CROSS technique. They were assessed clinically by the investigator and an independent physician by the scar counting method and digital photography at bi-weekly intervals. Local anesthetics or sedation were not used and patients were comfortable during the procedure. TCA 100% was made to order by a local pharmacy. To begin with, all the ice pick acne scars were marked with a pen and counted, followed by facial cleansing with soap and water and degreasing with acetone. The skin was stretched to reach the bottom of the scar and 100% TCA was then focally applied by pressing hard on the entire depressed area of atrophic acne scars using a tooth pick, taking care to avoid spillage to the surrounding skin [Figure 1]. The skin was kept stretched and monitored carefully until a refrigerator ‘frosted’ appearance after a single application was seen. Frosting was generally seen in 10 – 15 seconds and was a result of the coagulation of epidermal and dermal proteins and was used mainly to monitor the peel depth. The other types of atrophic acne scars including boxcar and rolling scars were not treated. A sunscreen was advised till the crusts were detached and continued till the end of the study period. No oral antibiotics were given. The application of makeup for camouflage was allowed. One week after CROSS, tretinoin cream 0.025% was reintroduced at night in all the patients. The procedure was repeated every two weeks for four sittings. The depth, appearance and number of acne scars were noted on each visit. The improvement was interpreted as excellent if > 70% reduction was observed, good if 50 – 70%, fair if 30 – 50% and poor if < 30% improvement was observed, according to a four-point scale. Side effects of therapy in the form of local burning, erythema, stinging sensation, temporary or prolonged hyperpigmentation and hypopigmentation were recorded. Further a statistical package of SPSSIO version was used for analysis. Patients were further called for follow up at intervals of three months and six months after the last treatment.

## RESULTS

**Figure 2:**
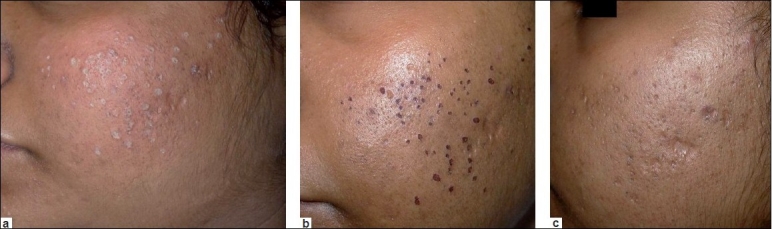
(a) Frosting with TCA 100%; (b) Crust formation on the third day; (c) Healing without any PIH on the seventh day in type V skin

**Figure 3:**
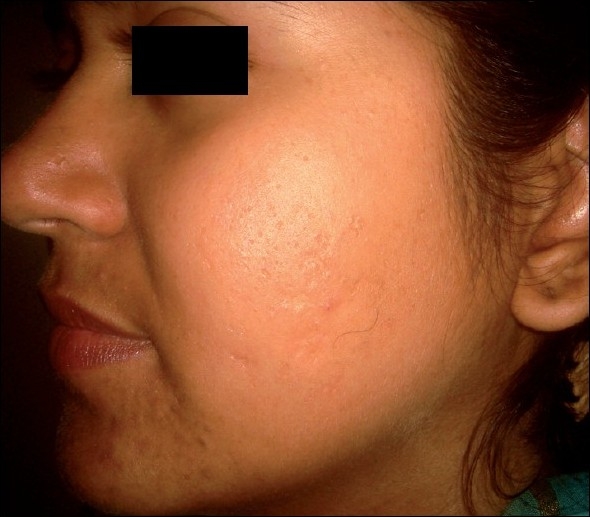
Excellent results 3 months after the last treatment

**Figure 4:**
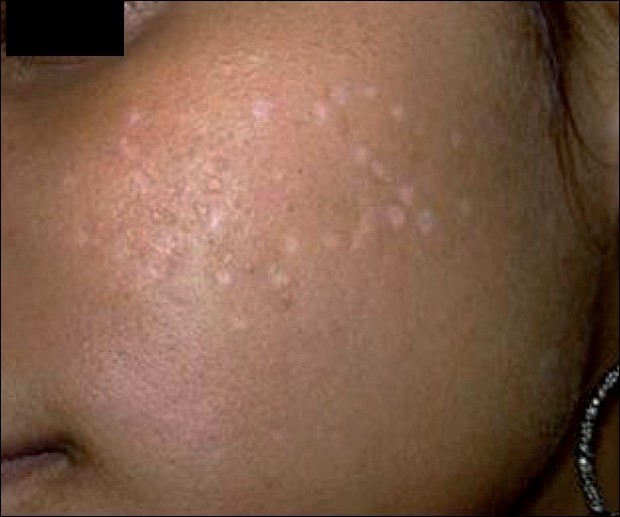
Transient hypopigmentation

Out of 12 patients, 10 patients completed the follow-up period of six months and were included in the study. Immediately on application, the patients experienced mild burning, which was well-tolerated. This was followed by frosting, erythema and oedema, that decreased in four to six hours. Crust formation was observed on the next day, which subsided by three to four days in most patients. [Figures 2 a, b and c]. In one patient crusting was observed for seven days. Eight patients (80%) showed excellent improvement [Figure 3], while two patients (20%) showed good results after four sessions [Table 1]. One patient had transient hypopigmentation that lasted for six days [Figure 4]. There were no major adverse effects observed such as post inflammatory hyperpigmentation, persistent erythema, herpes labialis flare-up, scarring, or keloid formation in any of the cases. The feedback from the patients was compared with the investigator results and observed to correlate in all. At three months of follow up, one out of ten patients observed a reduced effect and noted a decrease in improvement with no further improvement even at the six-month of follow-up. This patient had multiple ice pick scars that were close to each other. The other patients continued to show improvement.

**Table 1:** Assessment of results

Interpretation	Improvement (%)	No. of patients N = 10
Excellent	> 70	8 (80)
Good	50 – 70	2 (20)
Fair	30 – 50	-
Poor	< 30	-

Figures in parenthesis are in percentage

## DISCUSSION

Treatment of post acne scars is still a therapeutic challenge and requires a combination of many techniques such as subcision, punch excision, punch grafting, dermabrasion or laser resurfacing, chemical peels and the use of dermal fillers for the various types of scars.[6] Management of atrophic ice pick acne scarring is even more difficult as the scars extend deep into the dermis, even up to the subcutaneous tissue. Treatment options such as laser resurfacing or dermabrasion are hence not always successful. Besides, they are also associated with considerable morbidity and invariably prolonged downtime.[2]

Application of the caustic agent TCA to the skin in the concentration of 90% causes precipitation of proteins, coagulative necrosis of cells in the epidermis and necrosis of collagen in the papillary to upper reticular dermis as shown by Brodland and coworkers on porcine skin.[7] Yug *et al*,[8] have shown that dermal collagen remodelling may continue for several months. There is an increase in the dermal volume, as increased collagen production, glycosaminoglycan, and elastin fragmentation and reorganization is seen. Healing is more rapid and associated with a lower complication rate in the CROSS technique as the adjacent normal tissue and adnexal structures are spared.

Lee *et al*,[5] reported that 27 of 33 patients (82% of the 65% TCA group) and 30 of 32 patients (94% of the 100% TCA group) experienced a good clinical response. A better and faster response was seen in the 100% TCA group. All patients in the 100% TCA group who received five or six courses of treatment at monthly intervals showed excellent results. There were no cases of significant complication in individuals with darker skin, which was totally consistent with our study. One patient in our study had transient hypopigmentation that lasted for seven days. The results were achieved much earlier in our study; at the end of four sessions itself, in comparison to the previous studies.[5–9] A reduced effect and decrease in improvement after three months and six months of follow-up in one patient was probably due to reduction in dermal edema and delayed or poor collagenisation.

In a comparative split face study of 100% TCA CROSS applied twice at 12-week intervals on one side with a 1,550 nm Er : Glass fractional laser applied thrice at six-week intervals on the other side, showed that the CROSS technique was better for ice pick scars, but the fractional Er : Glass laser was more effective for rolling scars.[10] Although pain was more with Er : Glass laser, the downtime was less with the laser as compared to the CROSS technique. Another pilot study combined three modalities; the CROSS technique (called dot peeling in the study), subcision and fractional laser resurfacing to treat atrophic acne scars in 10 patients.[11] The rationale of this combination was to treat the ice pick scar with TCA, improve the texture of the skin and shallow scars with a 1550 nm fractional Er : glass laser, which had limitations in affecting ice pick and boxcar scars and treating wide, depressed boxcar or rolling scars with subcision. Acne scar severity scores decreased by a mean of 55.3%. In this study TCA was applied twice at an interval of two to three months. Cho *et al*,[12] treated 12 patients with atrophic acne scars and enlarged pores with a 1550 nm fractional Er: Glass laser for three sessions at monthly intervals under topical anaesthesia. They reported that three patients (25%) had 76 – 100% improvement in acne scars, and 5 (41.6%) had 51 – 75% improvement, while two patients each (16.7%) had moderate and minimal to no improvement. Thus, although the results were modest, with fractional lasers, they could be considered as a treatment option.

The results of our study indicated that higher treatment frequency of CROSS application improved the therapeutic effect and shortened the duration of treatment, without significant side effects. Furthermore, priming the skin for two weeks before the procedure helped in reducing the incidence of post inflammatory hyperpigmentation that was expected after using high concentrations of TCA on dark skins.

## CONCLUSION

CROSS technique with 100% TCA is a safe, costeffective, minimally invasive technique, with good efficacy, for the cosmetic management of ice pick acne scarring that can be used safely on darker skin Fitzpatrick (IV, V)-type individuals. However, in some patients early improvement may be due to dermal edema that leads to partial effacement of the scars. Decrease in efficacy at three and six months of follow up may be due to delayed collagenisation that may not be optimal at six months. Further studies in a larger number of patients, preferably with quantitative and qualitative histopathological analysis is indicated, to evaluate and validate the long-term results of this technique.
